# *Helicobacter pylori* Outer Membrane Vesicle Proteins Induce Human Eosinophil Degranulation via a *β*2 Integrin CD11/CD18- and ICAM-1-Dependent Mechanism

**DOI:** 10.1155/2015/301716

**Published:** 2015-03-04

**Authors:** Su Hyuk Ko, Jong Ik Jeon, Young-Jeon Kim, Ho Joo Yoon, Hyeyoung Kim, Nayoung Kim, Joo Sung Kim, Jung Mogg Kim

**Affiliations:** ^1^Department of Microbiology and Department of Biomedical Science, Hanyang University College of Medicine and Graduate School of Biomedical Science and Engineering, Sungdong-gu, Seoul 133-791, Republic of Korea; ^2^Department of Biotechnology, Joongbu University, Choongnam, Republic of Korea; ^3^Department of Internal Medicine, Hanyang University College of Medicine, Seoul, Republic of Korea; ^4^Department of Food and Nutrition, Brain Korea 21 PLUS Project, College of Human Ecology, Yonsei University, Seoul, Republic of Korea; ^5^Department of Internal Medicine, Seoul National University Bundang Hospital, Seongnam, Republic of Korea; ^6^Department of Internal Medicine, Seoul National University College of Medicine, Seoul, Republic of Korea

## Abstract

Eosinophil cationic protein (ECP), a cytotoxic protein contained in eosinophils granules, can contribute to various inflammatory responses. Although* Helicobacter pylori* infection increases infiltration of eosinophils, the mechanisms of eosinophil degranulation by* H. pylori* infection are largely unknown. The goal of this study was to investigate the role of* H. pylori* outer membrane vesicles (OMVs) in modulating eosinophil degranulation. We found that eosinophils treated with* H. pylori* OMVs released significantly more ECP compared with untreated controls. In addition, eosinophils cocultured with OMV-preexposed primary gastric epithelial cells exhibited significantly increased ECP release. Similarly, eosinophils cocultured with culture supernatant (CM) from primary gastric epithelial cells exposed to OMVs (OMV-CM) released significantly higher amounts of ECP compared with eosinophils cocultured with CM from unexposed control cells. Furthermore, OMVs and OMV-CM both induced the upregulation of ICAM-1 on gastric epithelial cells and *β*2 integrin CD11b on eosinophils. In addition, both transduction of* ICAM-1* shRNA into gastric epithelial cells and treatment with neutralizing mAbs to CD18 significantly decreased OMV-mediated or OMV-CM-mediated release of ECP. These results suggest that the eosinophil degranulation response to* H. pylori* OMVs occurs via a mechanism that is dependent on both *β*2 integrin CD11/CD18 and ICAM-1.

## 1. Introduction

The gastric pathogen* Helicobacter pylori* is a causative agent of diseases such as chronic gastritis, peptic ulcers, gastric cancers, and gastric mucosa-associated lymphoid tissue (MALT) lymphoma. Some studies have obtained evidence to support the coexistence of* H. pylori* infection and eosinophilic gastritis [1–4]; in one of these studies, the severity of chronic gastritis was even shown to be significantly correlated with the eosinophil score [1]. After clearance of* H. pylori*, the numbers of infiltrated eosinophils decreased slowly and remain elevated even at one year after treatment [2]. In addition, chronic gastritis induced by* H. pylori* infection also results in increased infiltration of eosinophils, which has been proposed to mediate pathogenic effects in* H. pylori*-infected patients with chronic gastric disorders [5]. Being consistent with this hypothesis, exposure of gastric epithelial cells to* H. pylori* for 24 h can increase production of eosinophil-migrating chemokines such as CCL2 (monocyte chemotactic protein-1, MCP-1), CCL5 (regulated on activation, normal T cell expressed and secreted, RANTES), and granulocyte-macrophage colony-stimulating factor (GM-CSF) [6].

Eosinophils are bone marrow-derived granulocytes that have specific granules containing large amounts of toxic materials. The activation of eosinophils results in their degranulation, an upregulation in cytokine production, and an increase of IgE production. The preformed granules within eosinophils contain four major cationic proteins that are cytotoxic: eosinophil peroxidase (EPO), eosinophilic cationic protein (ECP), eosinophil-derived neurotoxin (EDN), and major basic protein (MBP) [7]. Since chronic gastritis induced by* H. pylori* infection has been shown to result in increased eosinophil infiltration, and since infiltrated eosinophils may be associated with pathogenic effects in* H. pylori*-infected chronic gastritis [5, 8], degranulation of the infiltrated eosinophils may exert tissue damage. However, the precise cellular and molecular mechanisms of eosinophil degranulation in response to* H. pylori* infection are presently unclear.

The majority of* H. pylori* bacteria in the stomach remain unattached to the surface epithelium, even though the bacteria are able to adhere to gastric epithelial cells [9]. These bacteria release portions of their outer membrane in vesicular form; these compartments are referred to as outer membrane vesicles (OMVs). Since OMVs are derived from the outer membrane of the cell, they contain many surface elements of the bacterium, such as lipopolysaccharide (LPS) and outer membrane proteins. In addition, nonadherent* H. pylori* have been shown to release OMVs* in situ*; moreover, these vesicles can act as a vehicle for vacuolating cytotoxin (VacA) [10]. In addition,* H. pylori* OMV can be internalized to gastric epithelial cells [10, 11]. After internalization, OMVs have been hypothesized to modulate gastric epithelial cell proliferation, induce apoptosis, stimulate secretion of interleukin (IL)-8, and increase micronucleus formation (reviewed in [10]). Therefore,* H. pylori*-derived OMVs may contribute to the* H. pylori*-induced pathogenic effects that have been observed in the stomach.

Eosinophil adhesion to bronchial epithelial cells can be an important signal for the activation and degranulation of eosinophils [12]. In addition, exposure of gastric epithelial cells to* H. pylori* can produce significant amounts of eosinophil-migrating chemokines [6]. Based on these findings, we hypothesized that eosinophil adhesion to gastric epithelial cells may be a signal for the activation and degranulation of eosinophils. In this study, we investigated the role of OMVs in human eosinophil effector functions and found that* H. pylori* OMVs and OMV-preexposed gastric epithelial cells could trigger the release of granule proteins from human eosinophils via a mechanism involving intercellular adhesion molecule-1 (ICAM-1) and *β*2 integrin CD11/CD18.

## 2. Materials and Methods

### 2.1. Reagents

LPS-free fetal bovine serum (FBS), antibiotics, L-glutamine, Trizol, and Ca^2+^ and Mg^2+^-free Hank's balanced salt solution (HBSS) were obtained from GIBCO BRL (Gaithersburg, MD, USA). Brucella broth was purchased from Becton, Dickinson, and Company (Sparks, MD, USA). RNase A, DNase II, proteinase K, dithiothreitol (DTT), bovine serum albumin (BSA), Histopaque, RBC lysis buffer, and RPMI-1640 medium were purchased from Sigma-Aldrich Chemical Co. (St. Louis, MO, USA). Fluorescein isothiocyanate- (FITC-) conjugated monoclonal antibodies (mAbs) against ICAM-1, CD11a, CD11b, and CD11c for flow cytometry were purchased from Santa Cruz Biotechnology (Santa Cruz, CA, USA). Anti-human mAb to CD18 for neutralization was obtained from eBioscience (clone R3.3, San Diego, CA, USA).

### 2.2. *H. pylori* Strains

The* H. pylori* strain 60190 (ATCC 49503, CagA^+^,* vacA* s1a/m1) was used for the purification of OMVs. The CagA^−^ isogenic mutant, VacA^−^ isogenic mutant, and PicB^−^/CagE^−^ isogenic mutant were obtained from Dr. Yong Chan Lee (Yonsei University College of Medicine, Seoul, Korea) with the kind permission of Dr. Martin J. Blaser (New York University Langone Medical Center, NY, USA). All* H. pylori* strains were cultured under microaerophilic conditions (5% O_2_, 10% CO_2_, and 85% N_2_).

### 2.3. Preparation of* H. pylori* OMVs, LPS, and VacA

*H. pylori* OMVs were prepared according to a previously described protocol [9]. Briefly,* H. pylori* strain 60190 (ATCC 49503, CagA^+^,* vacA* s1a/m1) was grown in 2.8% (wt/vol) Brucella broth supplemented with 5% FBS at 37°C under microaerobic conditions with constant rotation (120 rpm). After 72 h of incubation, bacteria were removed by two centrifugations (12,000 ×g, 15 min, 4°C), and the final supernatants ultracentrifuged (200,000 ×g, 2 h, 4°C) to recover OMVs. After three washes in phosphate-buffered saline (PBS), the OMVs were stored at −20°C until required. The protein concentrations of the OMV preparations were determined by the Bradford method (Bio-Rad, Hercules, CA, USA). Medium without bacteria were used as a control.

Extraction of* H. pylori* LPS was performed using a conventional hot phenol-water treatment. This crude LPS extract was subsequently purified by enzymatic treatments with RNase A, DNase II, and proteinase K as previously described [9, 13].

For purification of VacA proteins, the VacA-producing* H. pylori* strain 60190 was grown in sulfite-free Brucella broth containing 0.5% charcoal (untreated, granular 8–20 mesh) at 37°C under microaerophilic conditions. VacA was purified from broth culture supernatants according to previously described methods [14–17]. Immediately before use, the purified VacA protein was activated by the addition of 250 mM HCl, until the pH reached 2.0. NH_4_Cl (5 mM) was also added to the medium to enhance VacA activity.

### 2.4. Isolation of Peripheral Blood Eosinophils and Primary Human Gastric Epithelial Cells

Eosinophils were isolated from the peripheral blood of human volunteers using a magnetic cell separation system (Miltenyi Biotec, Bergisch Gladbach, Germany) as previously described [14, 15, 18]. The Hanyang University College of Medicine Review Board approved the protocol that was used to obtain blood from human volunteers. In brief, venous blood that had been anticoagulated with heparin was diluted with piperazine-N,N9-bis (2-ethanesulfonic acid) (PIPES) buffer (25 mM PIPES, 50 mM NaCl, 5 mM KCl, 25 mM NaOH, and 5.4 mM glucose, pH 7.4) at a 1 : 1 ratio. Diluted blood was layered onto a Histopaque solution (density, 1.083 g/mL) and centrifuged at 100 ×g at 4°C for 30 min. The supernatant and mononuclear cells at the interface were both carefully removed. Erythrocytes present in the sediment were lysed by exposure to RBC lysis buffer. Isolated granulocytes were then washed with PIPES buffer containing 1% FBS, and an approximately equal volume of anti-CD16 Ab-conjugated magnetic particles (Miltenyi Biotec) was added to the cell pellet. After 30 min of incubation on ice, cells were loaded onto the separation column, which was positioned in the magnetic cell separation system with a magnetic field. Cells were eluted with PIPES buffer containing 1% FBS [14, 15, 18]. The purity of PBMCs, as determined by flow cytometry using anti-CD14 Abs, was >95%. The purity of eosinophils as assessed by Randolph's stain was >98%. Purified eosinophils were used immediately for experiments using RPMI 1640 medium.

Primary human gastric epithelial cells were isolated from the apparently normal mucosa of surgically resected stomachs obtained from patients with gastric cancer, as previously described [19]. This study was approved by the Ethics Committee of Seoul National University Hospital. After the surgical specimens were washed twice in HBSS, the epithelia were removed by scraping the surfaces with a glass slide. The scraped tissue was cut into small sections using a razor blade and then washed at room temperature with 10 mM DTT in HBSS for 30 min. Tissue sections were then subjected to two additional 90 min washes in 1 mM EDTA. Cells liberated from both washes were harvested by centrifugation at 500 ×g for 5 min at room temperature and then incubated with 3 mg/mL dispase and 1 mg/mL DNase at 37°C for 30 min. Cells were harvested by centrifugation and then further purified using a Percoll density gradient (Pharmacia Biotech, Uppsala, Sweden), in which the intestinal epithelial cells are located at the 0–30% layer interface. After centrifugation for 20 min at 300 ×g, purified epithelial cells were collected. The isolated cells were cultured at a density of 2 × 10^6^ cells/mL in RPMI 1640 medium supplemented with 10% FBS, 2 mM glutamine, and antibiotics (100 U/mL of penicillin and 100 *μ*g/mL of streptomycin) [19]. Epithelial cell preparations contained less than 5% contamination from B cells or monocytes/macrophages, as assessed by flow cytometry using CD19/20 and CD14 as purity markers.

### 2.5. Preparation of Culture Supernatant from Gastric Epithelial Cells Stimulated with* H. pylori* OMVs

For preparation of culture supernatant (CM) from primary human gastric epithelial cells stimulated with* H. pylori* OMV, gastric epithelial cells were exposed to OMVs (200 *μ*g/mL) for 24 h and then the CM was collected. To minimize the effect of any potentially contaminating OMVs, the CM was filtered using two sequential filters, first a 0.1 *μ*m filter (Merck Millipore, Billerica, MA, USA), and then an Amicon Ultra-4 centrifugal filter unit with a molecular weight of 100 kDa (Merck Millipore). The resultant filtered CM was designated as “OMV-CM.” The medium obtained from primary human gastric epithelial cells cultured for 24 h in the absence of OMVs was designated as “control-CM.” In some experiments, primary human gastric epithelial cells were exposed to OMV-CM (50% v/v) or control-CM (50% v/v) for 24 h and then washed in PBS two times. After these washes, human eosinophils were cocultured with either OMV-CM-exposed or control-CM-exposed gastric epithelial cells for 24 h.

### 2.6. ECP Determination for Eosinophil Degranulation

To measure the extent of ECP release, freshly isolated eosinophils (1 × 10^5^ cells/0.5 mL) in 24-well plates were incubated with the indicated concentrations of OMVs for 24 h at 37°C in 5% CO_2_. After this incubation, the culture supernatants were collected. The amounts of ECP in the supernatants were determined using an ELISA kit (Antibodies-online, Inc., Atlanta, GA, USA) according to the manufacturer's instructions. Each experiment was performed in triplicate wells.

To determine the role of gastric epithelial cells in eosinophil degranulation, epithelial cells were incubated with OMVs, OMV-CM, or control-CM in 24-well plates. At 24 h, the medium was removed and 0.5 mL of an eosinophil suspension (1 × 10^5^ cells) was added to each well. In some experiments, mouse anti-human mAbs against CD11b or ICAM-1, or mouse IgG isotype, as a control, were added to the wells at the beginning of the coculture.

### 2.7. Flow Cytometric Analysis

To quantitate the surface expression of ICAM-1 on gastric epithelial cells and the expression of CD11a, CD11b, and CD11c on eosinophils, cells were twice washed with cold Ca^2+^ and Mg^2+^-free HBSS. Resuspended cells were centrifuged at 200 ×g for 5 min at 4°C and then washed with HBSS containing 0.5% BSA. Washed cells were transferred to flow cytometry tubes and centrifuged at 500 ×g for 3 min at 4°C, after which the supernatants were discarded. The cells were then fixed in formaldehyde (4%) for 10 min. Thereafter, cells were incubated with FITC-conjugated anti-human mAb in 0.5% BSA. After 1 h, cells were washed twice with cold HBSS containing 0.5% BSA. Immunostained cells were then analyzed by flow cytometry (FACSCalibur cytometer, Becton Dickinson and Company, San Jose, CA, USA). Ten thousand cells were analyzed per sample; the expression level of each molecule is expressed as the mean fluorescence intensity (MFI) [20, 21].

### 2.8. Quantitative RT-PCR Analysis

Freshly isolated human eosinophils were stimulated with OMV or OMV-CM, after which total cellular RNA was extracted using Trizol. Expressed mRNA transcript levels were measured by quantitative RT-PCR using internal standards. The oligonucleotide primers used for PCR amplification and the sizes of the PCR products obtained from target cellular RNA and synthetic standard RNA have been previously described [21, 22]. PCR amplification consisted of 35 cycles of a 1 min denaturation step at 95°C, a 2.5 min annealing step, and an extension step at either 65°C (ICAM-1) or 72°C (*β*-actin). The sizes of the PCR products generated from standard RNAs for human ICAM-1 and *β*-actin were 480 bp and 520 bp, respectively.

### 2.9. Transduction Assay

Lentiviral particles containing short hairpin RNA (shRNA) against* ICAM-1* or control shRNA were purchased from Santa Cruz Biotechnology. Transduction of gastric epithelial cells with lentiviral particles was performed according to the manufacturer's instructions [23].

### 2.10. Statistical Analyses

Data are presented as mean ± standard deviation (SD) or mean ± standard error of the means (SEM). Statistical evaluation of data was accomplished by using a Student's* t*-test to compare two samples or ANOVA for more than two samples.* P* values <0.05 were considered significant.

## 3. Results

### 3.1. Effect of* H. pylori* OMVs on ECP Release

**Figure 1:**
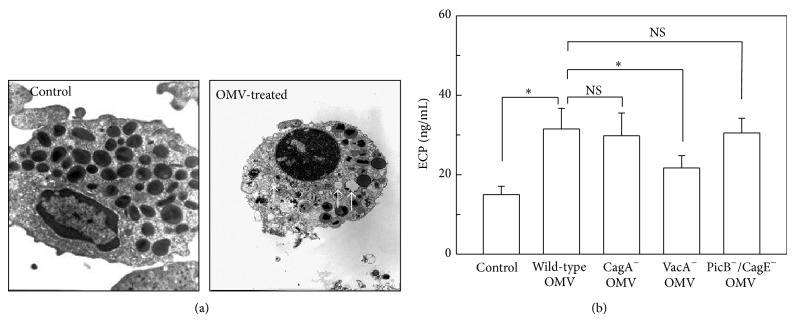
Degranulation of eosinophils stimulated with* H. pylori* OMVs. (a) Freshly isolated human eosinophils were stimulated with* H. pylori* OMVs (200 *μ*g/mL) for 24 h. Cells were analyzed by TEM (×12,000). OMV-treated cells exhibited cytoplasmic degranulation (arrowhead). (b) Human eosinophils were stimulated with OMVs (200 *μ*g/mL) obtained from wild-type* H. pylori* or the indicated isogenic mutants for 24 h. The concentrations of ECP in the culture supernatants were measured by ELISA (mean ± SEM, *n* = 5). ^*^ *P* < 0.05 control; NS, statistically nonsignificant.

We first examined whether OMVs obtained from* H. pylori* could induce eosinophil degranulation. Freshly isolated human eosinophils were treated with OMVs and then analyzed by transmission electron microscopy (TEM). As shown in Figure 1(a), eosinophils treated with* H. pylori* OMVs for 24 h exhibited cytoplasmic degranulation.

To validate this result with a complementary approach, the levels of ECP in the supernatants of treated cells were measured by ELISA. Eosinophils stimulated with* H. pylori* OMVs for 24 h released more ECP than did unstimulated eosinophils (Figure 1(b)). Moreover, OMVs obtained from a VacA-negative isogenic mutant strain induced less ECP release than OMVs obtained from a wild-type strain. However, no significant difference was observed regarding the induction of ECP release when OMVs were obtained from a CagA-negative isogenic mutant strain, a PicB^−^/CagE-negative isogenic mutant strain, or a wild-type strain.

**Figure 2:**
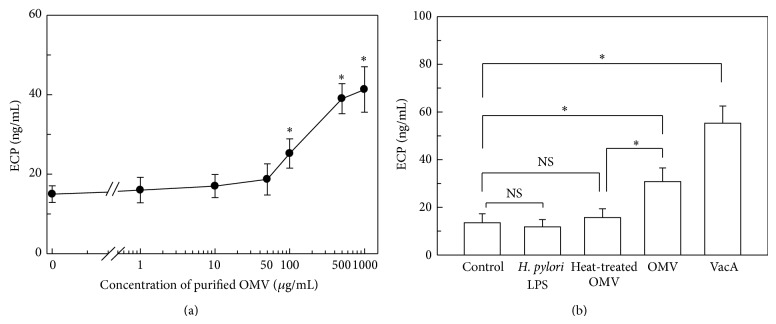
ECP release from eosinophils stimulated with intact* H. pylori* OMVs or individual components of OMVs. (a) ECP release is induced in a dose-dependent manner by* H. pylori* OMVs. Freshly isolated human eosinophils were treated with the indicated concentrations of* H. pylori* OMVs for 24 h. (b) Human eosinophils were treated with* H. pylori* LPS (1,000 ng/mL), heat-treated OMVs (200 *μ*g/mL), intact OMVs (200 *μ*g/mL), or purified VacA (1,000 ng/mL) for 24 h. The concentration of ECP in each culture supernatant was determined by ELISA (mean ± SEM, *n* = 5). ^*^ *P* < 0.05; NS, statistically nonsignificant.

The magnitude of ECP release was dependent on the concentration of OMVs. As shown in Figure 2(a), the amount of ECP released by the stimulated eosinophils increased as the concentration of OMVs increased. The concentration of OMVs that yielded a half-maximal response (EC50) was calculated to be 142.8 *μ*g/mL by SigmaPlot 10.0 software (Systat Software Inc., San Jose, CA, USA). Based on these results, OMVs were used 200 *μ*g/mL for all stimulation in the present study.

OMVs are known to contain many surface elements of the bacterial cell wall such as LPS [10, 24]. To investigate the role of* H. pylori* LPS on ECP release,* H. pylori* LPS was added to freshly isolated human eosinophils. In these experiments,* H. pylori* LPS did not induce a significant release of ECP from eosinophils compared with unstimulated control cells (Figure 2(b)). When OMVs were heat inactivated at 60°C for 30 min, the amount of ECP released from stimulated eosinophils was significantly decreased compared with the amount released from eosinophils stimulated with intact OMVs. This result suggests that the eosinophil degranulation may be mediated by protein components of the OMVs. A similar experiment was performed to determine whether purified* H. pylori* VacA affects ECP released from eosinophils. As shown in Figure 2(b), VacA significantly enhanced ECP release from eosinophils. These results suggest that the direct contact of eosinophils with either OMV-resident proteins or VacA induces degranulation during* H. pylori* infection.

### 3.2. ECP Release in Coculture of Eosinophils and* H. pylori* OMV-Exposed Gastric Epithelial Cells

**Figure 3:**
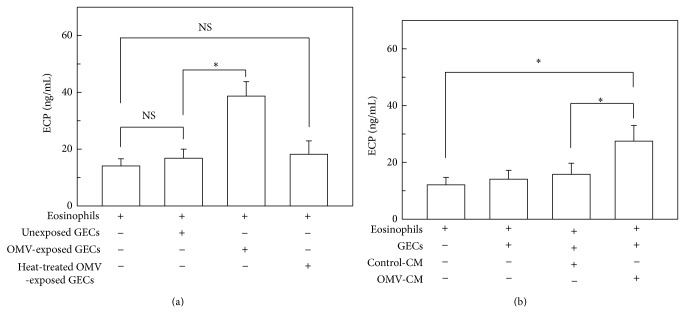
Effects of* H. pylori* OMV-exposed gastric epithelial cells on ECP released from eosinophils. (a) Primary human gastric epithelial cells (GECs) were exposed to either OMVs (200 *μ*g/mL) or heat-treated OMVs (200 *μ*g/mL) for 24 h and then washed twice in PBS. Human eosinophils were cocultured with either OMV-exposed or -unexposed GECs for 24 h. The concentrations of ECP in the culture supernatants were measured by ELISA (mean ± SEM, *n* = 5). ^*^ *P* < 0.05; NS, statistically nonsignificant. (b) Primary human GECs were cultured for 24 h in either the presence or absence of OMVs (200 *μ*g/mL). Culture supernatants were then collected as described in Materials and Methods. GECs were stimulated with control-CM (50% v/v) or OMV-CM (50% v/v) for 24 h and then washed twice in PBS. Finally, control-CM-exposed or OMV-CM-exposed GECs were cocultured with human eosinophils for 24 h. The concentrations of ECP in the culture supernatants were measured by ELISA (mean ± SEM, *n* = 5). ^*^ *P* < 0.05.

We next asked whether adhesion of eosinophils to gastric epithelial cells could induce eosinophil degranulation. In these experiments, an* in vitro* coculture model of eosinophils and primary human gastric epithelial cells was employed. As shown in Figure 3(a), eosinophils cocultured with unexposed gastric epithelial cells for 24 h released the almost same amount of ECP as eosinophils cultured in the absence of gastric epithelial cells. In a concurrent experiment, gastric epithelial cells were exposed to OMVs for 24 h, after which cells were cocultured with eosinophils. The amount of ECP released from eosinophils cocultured with OMV-exposed gastric epithelial cells was higher than the amount released from eosinophils cocultured with unexposed gastric epithelial cells. When gastric epithelial cells were stimulated with heat-inactivated OMVs (60°C for 30 min) and then cocultured with eosinophils, the amount of ECP release was slightly increased; however, this difference was not significant. These results suggest that the direct contact of eosinophils with OMV-exposed gastric epithelial cells can induce eosinophil degranulation.

During microbial infection, uninfected bystander epithelial cells can be exposed to a number of soluble mediators produced by adjacent infected cells [25]. In* H. pylori* infection, gastric epithelial cells constitute the initial sites of interaction of the host with* H. pylori*. Interestingly, intestinal epithelial cells appear to rapidly contact and respond to bacterial-derived OMVs. Therefore, we investigated whether eosinophil degranulation was induced by CM obtained from primary gastric epithelial cells exposed to OMVs (OMV-CM) for 24 h. Gastric epithelial cells were incubated with OMV-CM or control-CM for 24 h, after which time cells were cocultured with eosinophils. As shown in Figure 3(b), eosinophils cocultured with gastric epithelial cells that had been pretreated with OMV-CM released significantly higher amounts of ECP than eosinophils cocultured with gastric epithelial cells that had been pretreated with control-CM. These results suggest that the interaction between eosinophils and bystander gastric epithelial cells, which have likely been stimulated by soluble mediators produced from OMV-exposed cells, may also be involved in the degranulation response to* H. pylori* infection.

### 3.3. Expression of ICAM-1 on Gastric Epithelial Cells and Expression of CD11b on Eosinophils Are Both Upregulated by* H. pylori* OMVs and the Culture Supernatant from OMV-Exposed Gastric Epithelial Cells

**Figure 4:**
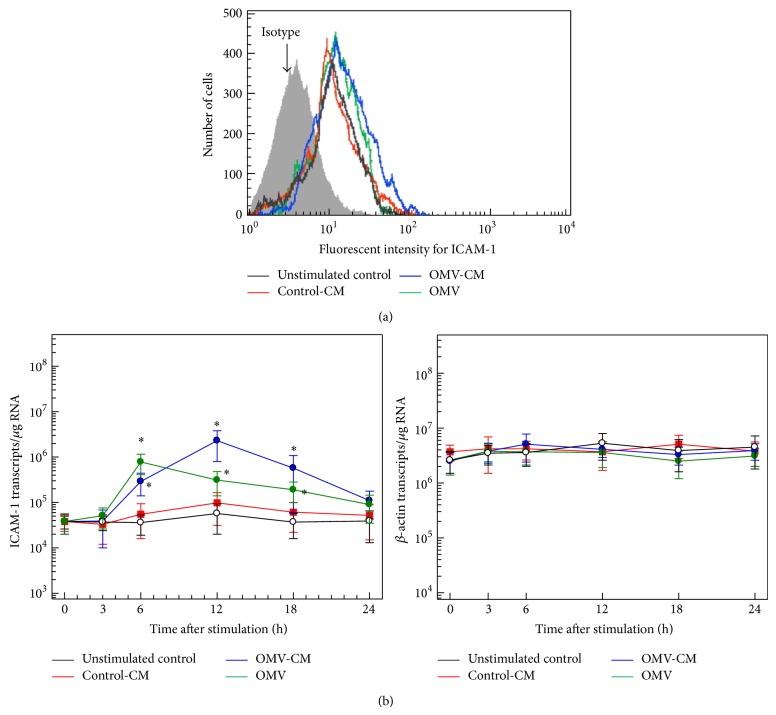
ICAM-1 expression in gastric epithelial cells stimulated with* H. pylori* OMVs or OMV-induced culture supernatant. (a) OMV-CM and control-CM were prepared as described in Materials and Methods. Primary human gastric epithelial cells were stimulated with OMVs (200 *μ*g/mL), OMV-CM (50% v/v), or control CM (50% v/v) for 24 h. Cells were stained with a mAb against ICAM-1 and then analyzed using flow cytometry. Results are representative of more than five independent experiments. (b) Time course of ICAM-1 mRNA expression in gastric epithelial cells after stimulation with OMVs, OMV-CM, or control-CM. Cells were stimulated with OMVs (200 *μ*g/mL), OMV-CM (50% v/v), or control-CM (50% v/v) for the indicated periods of time. The expression levels of ICAM-1 and *β*-actin mRNA were analyzed by quantitative RT-PCR using standard RNAs. Values are expressed as mean ± SD (*n* = 5). Asterisks indicate statistical significance after comparison with unstimulated controls (*P* < 0.05).

Integrins of the *β*2 family (CD11/CD18) play a critical role in eosinophil degranulation, which is induced by stimuli such as viral infection and cytokines [25, 26]. ICAM-1 is one of the predominant cell adhesion molecules expressed in response to chronic* H. pylori* infection; moreover, increased expression of ICAM-1 has been linked to massive infiltration of inflammatory cells that express LFA-1 and Mac-1 [27]. In the present study, exposure of gastric epithelial cells to OMVs or OMV-CM appeared to increase ICAM-1 expression, as assessed by flow cytometric analysis (Figure 4(a)). These results were confirmed by quantitative RT-PCR analysis for ICAM-1 mRNA transcripts. As shown in Figure 4(b), primary gastric epithelial cells cocultured with OMVs or OMV-CM exhibited significantly increased ICAM-1 mRNA expression. However, the kinetics of mRNA upregulation were different; the levels of OMV-induced and OMV-CM-induced ICAM-1 mRNA transcripts reached their maximum levels after 6 h and 12 h, respectively, and returned to basal levels 24 h after stimulation.

**Figure 5:**
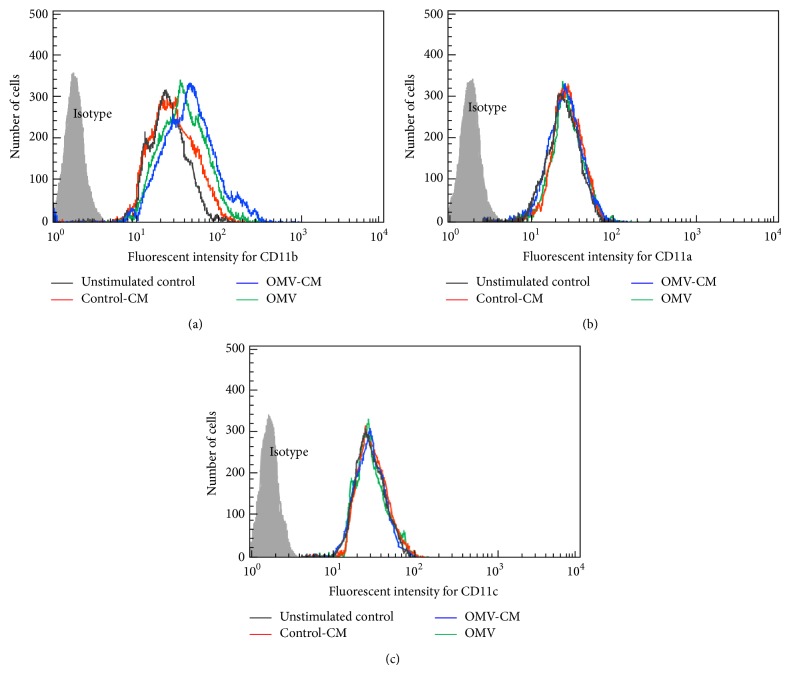
Increased CD11b expression in eosinophils stimulated with* H. pylori* OMVs or OMV-CM. Freshly isolated human eosinophils were stimulated with OMVs (200 *μ*g/mL), OMV-CM (50% v/v), or control-CM (50% v/v) for 24 h. Cells were stained with mAb against CD11b (a), CD11a (b), or CD11c (c) and then analyzed using flow cytometry. Results are representative of more than five independent experiments.

We next asked whether OMVs and/or OMV-CM influenced CD11 expression in eosinophils. Freshly isolated blood eosinophils are known to constitutively express CD11a, CD11b, and CD11c. We found that eosinophils stimulated with OMVs or OMV-CM exhibited increased CD11b expression compared with eosinophils stimulated with untreated CM, as assessed by flow cytometry (Figure 5(a)). However, eosinophils stimulated with OMVs or OMV-CM did not exhibit significant changes in the expression of surface CD11a or CD11c molecules compared with unstimulated control cells (Figures 5(b) and 5(c)).

### 3.4. ICAM-1 and CD11/CD18 Molecules Are Associated with ECP Released from Eosinophils Cocultured with Gastric Epithelial Cells

**Figure 6:**
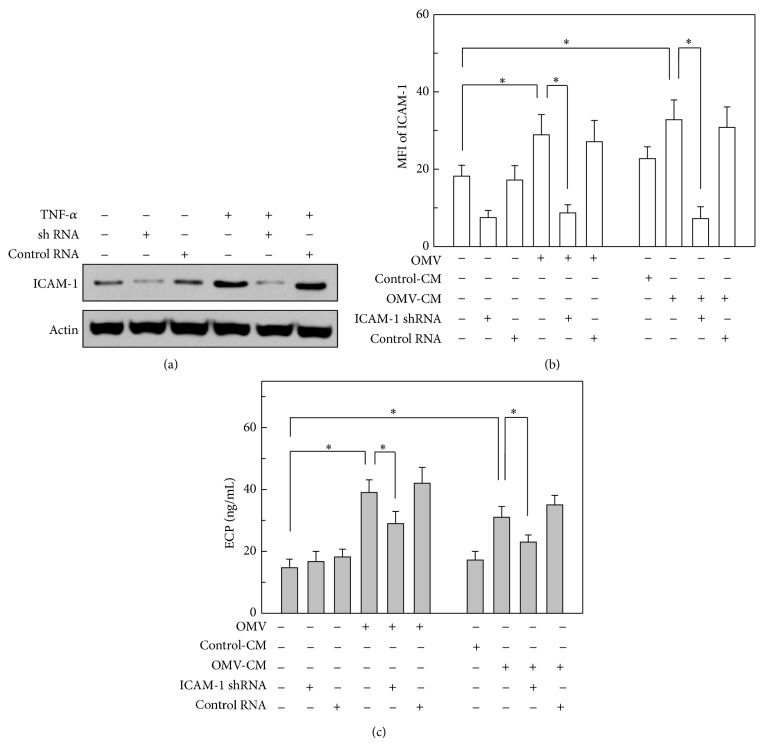
Relationship between ICAM-1 suppression and ECP release from eosinophils. (a) Primary human gastric epithelial cells were transduced with lentivirus harboring either shRNA directed against* ICAM-1* or control shRNA. Transduced cells were then stimulated with TNF-*α* (20 ng/mL) for 1 h. The cellular levels of ICAM-1 and actin were determined by immunoblot analysis. Results are representative of more than three independent experiments. (b) Transduced or nontransduced gastric epithelial cells were stimulated with OMVs (200 *μ*g/mL), OMV-CM (50% v/v), or control-CM (50% v/v) for 24 h. Cells were stained with a mAb against ICAM-1 and then analyzed by flow cytometry. Data are represented as MFI ± SEM (*n* = 5). (c) Transduced or nontransduced gastric epithelial cells were exposed to OMVs (200 *μ*g/mL) for 24 h and then washed twice in PBS. OMV-exposed gastric epithelial cells were cocultured with human eosinophils for 24 h (left panel). Transduced or nontransduced gastric epithelial cells were stimulated with control-CM (50% v/v) or OMV-CM (50% v/v) for 24 h and then washed twice in PBS. Finally, human eosinophils were cocultured with either control-CM-stimulated or OMV-CM-stimulated epithelial cells for 24 h (right panel). The concentration of ECP in each culture supernatant was measured by ELISA (mean ± SEM, *n* = 5). ^*^ *P* < 0.05.

Since ICAM-1 on gastric epithelial cells (Figure 4) and CD11b on eosinophils (Figure 5) were both upregulated in response to stimulation with either OMVs or OMV-CM, we next asked the roles of these adhesion molecules in eosinophil degranulation. To evaluate the extent of ICAM-1-dependent eosinophil degranulation, primary gastric epithelial cells were transduced with lentivirus harboring a shRNA sequence against* ICAM-1* to silence the expression of ICAM-1. As shown in Figure 6(a), shRNA delivered by lentiviral transduction effectively silenced the level of ICAM-1 expression in gastric epithelial cells; however, transduction with a lentivirus harboring control shRNA did not affect ICAM-1 expression upon TNF-*α*-stimulation. In this experimental system, transduced cells were stimulated with either OMVs or OMV-CM, and the surface expression of ICAM-1 molecules was analyzed using flow cytometry. As expected, cells transduced with lentivirus harboring shRNA against* ICAM-1* exhibited significantly reduced surface expression of ICAM-1; however, control lentivirus did not affect ICAM-1 expression (Figure 6(b)). In further experiments, transduced cells preincubated with OMVs or OMV-CM were cocultured with eosinophils. Eosinophils cocultured with nontransduced or control lentivirus-transduced cells, both of which had been preincubated with OMVs, exhibited significantly increased ECP release.

In addition, the amount of ECP released from eosinophils cocultured with epithelial cells that had been transduced with* ICAM-1* shRNA-harboring lentivirus was higher than the amount released from eosinophils cocultured with nontransduced or control lentivirus-transduced cells (Figure 6(c), left panel). Similar results were observed for gastric epithelial cells transduced with lentivirus upon OMV-CM stimulation (Figure 6(c), right panel).

**Figure 7:**
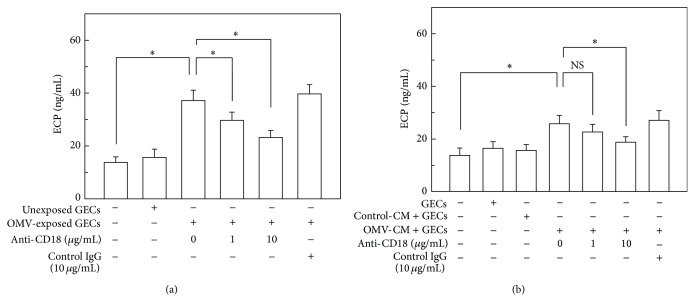
Effects of anti-CD18 mAb on ECP released from eosinophils. (a) Eosinophils were cocultured either with unexposed control cells or with OMV-exposed primary human gastric epithelial cells (GECs) for 24 h in the presence of either anti-CD18 mAb or mouse IgG isotype control Ab. The concentration of ECP in each culture supernatant was measured by ELISA (mean ± SEM, *n* = 5). (b) Human eosinophils were cocultured either with OMV-CM-exposed or control CM-exposed GECs for 24 h in the presence of either anti-CD18 mAb or mouse IgG isotype control Ab. The concentration of ECP in each culture supernatant was measured by ELISA (mean ± SEM, *n* = 5). ^*^ *P* < 0.05 compared with unstimulated control; NS, statistically nonsignificant.

We next investigated the effects of integrin *β*2 family members (CD11/CD18) on eosinophil degranulation. As shown in Figure 7(a), the addition of anti-CD18 mAb (clone CBRM1/5) significantly inhibited ECP release in a dose-dependent manner. In further experiments, the addition of anti-CD18 mAb also significantly reduced ECP release from eosinophils that had been cocultured with OMV-CM-stimulated gastric epithelial cells (Figure 7(b)).

## 4. Discussion

Eosinophil degranulation is a natural physiological phenomenon* in vivo*. However, excessive degranulation is known to cause epithelial damage via the release of cytotoxic granule proteins such as ECP. Since chronic gastritis induced by* H. pylori* infection results in increased infiltration of eosinophils, and since infiltrated eosinophils have also been hypothesized to mediate pathogenic effects in* H. pylori*-infected chronic gastritis [5], degranulation may play an important role in the pathogenesis of* H. pylori* infection. The present study demonstrated that the direct contact of human eosinophils with* H. pylori* OMVs or with gastric epithelial cells exposed to OMVs can induce excessive eosinophil degranulation.

Eosinophils begin life and reside in the bone marrow for 8 days while undergoing maturation. They subsequently relocate into the peripheral circulation for 8–12 h and finally traffic to specific tissues, predominantly the gastrointestinal tract, in which they reside for at least 1 week [7]. Eosinophils are predominantly tissue-dwelling cells with the high affinity for epithelial surfaces that interact with the external environment (e.g., skin, lung, and gastrointestinal tract) [7]. In the gastrointestinal tract, eosinophils customarily reside in the lamina propria [7]. In this regard, OMVs shed from* H. pylori* are likely to contact eosinophils resident in the lamina propria [28, 29]. Nevertheless, the cellular and molecular mechanisms of eosinophil degranulation have not been previously reported in the context of* H. pylori* infection.

It has become clear that gastric epithelial cells, which are known to be the main targets of* H. pylori* infection, are themselves able to initiate and modulate local inflammatory responses by releasing a variety of cytokines and inflammatory mediators. Therefore, our experimental approach was designed to mimic the* in vivo* interaction between the* H. pylori* OMV-exposed epithelium and eosinophils within the gastric mucosa. For this purpose, ECP release was investigated using cocultures of primary human gastric epithelial cells and eosinophils.

In the present study, eosinophils released significant amounts of ECP when they were exposed to OMVs, suggesting that the uptake of OMVs may lead to eosinophil degranulation. Exposure of eosinophils to OMVs obtained from a VacA-negative isogenic mutant strain induced less ECP release compared to OMVs from a wild-type strain. However, the absence of VacA just partially reduced the secretion of ECP, suggesting that additional factors may modulate eosinophil degranulation induced by OMV preparations. A recent study has demonstrated that Hp(2-20) peptides derived from* H. pylori* stimulated eosinophils migration through the interaction with formyl-peptide receptors [30]. Therefore, Hp(2-20) peptides derived from* H. pylori* may be involved in the ECP release in eosinophils stimulated with OMVs. Further studies are needed to investigate this possibility.

Coculture of eosinophils with OMV-exposed gastric epithelial cells also stimulated ECP release. Unexposed gastric epithelial cells also appear to induce eosinophil degranulation; however, this difference was not statistically significant. Interestingly, eosinophils cocultured with gastric epithelial cells induced a significant release of ECP proteins in the presence of CM obtained from OMV-exposed gastric epithelial cells (OMV-CM). These results indicate that factors for eosinophil degranulation may be acquired via the uptake of* H. pylori* OMVs by eosinophils and/or the direct contact of eosinophils with OMV-exposed or OMV-CM-exposed gastric epithelial cells.

Although adhesion studies were not performed in the present study, the addition of a neutralizing mAb to the CD18 surface molecules may inhibit eosinophil adhesion to gastric epithelial cells. The *β*2 integrins comprise a family containing three receptors: CD11a/CD18 (LFA-1), CD11b/CD18 (macrophage-1 antigen (Mac-1) or integrin *α*M*β*2), and CD11c/CD18 (p150/95). These receptors are heterodimers of two noncovalently linked polypeptide chains with distinct *α*-chains (CD11a, 11b, and 11c) and a common *β*2-chain (CD18) [31]. These integrins, which are expressed by eosinophils, can bind to adhesion molecules such as ICAM-1, which is present on vascular endothelial cells. In the present study, stimulation of eosinophils with OMVs or OMV-CM increased the surface expression of CD11b. In addition, exposure of gastric epithelial cells to OMVs or OMV-CM upregulated surface expression of ICAM-1. These results led us to form the hypothesis that* H. pylori* OMVs and/or OMV-CM may be involved in the adhesion of eosinophils to gastric epithelial cells, thereby leading to eosinophil degranulation.

With regard to eosinophil activation, our results indicate that OMV-induced or OMV-CM-induced ICAM-1 expression may be involved in eosinophil degranulation. In addition, the exposure of eosinophils to OMVs or OMV-CM induced the upregulation of CD11b, which is the *α*-chain of the *β*2 integrin CD11b/CD18. These molecules are involved in the specific adhesion of leukocytes to endothelial cells and extracellular matrices [32, 33]. Addition of anti-CD18 mAb significantly inhibited ECP released from eosinophils cocultured with gastric epithelial cells that had been stimulated with either OMVs- or OMV-CM. Therefore, OMV-induced or OMV-CM-induced eosinophil degranulation may be associated with a mechanism that is dependent on both *β*2 integrin CD11b/CD18 and ICAM-1.

Chemokine CCL5 (RANTES) is known to increase the adhesion avidity of *β*2 integrins in eosinophils [34].* H. pylori* infection has been shown to upregulate chemokine CCL5 in gastric epithelial cells as well as eosinophils [6, 14, 35, 36]. Considering our findings that OMV-CM induced a significant upregulation of CD11b, in addition to excessive degranulation, OMV-induced CM is likely to be a rich source of CC chemokines. In addition, OMV-induced CM may modulate adhesion and other functions of eosinophils by increasing both the number and the avidity of CD11b molecules on eosinophils. Therefore, CM released from OMV-exposed gastric epithelial cells may provide an appropriate milieu of inflammatory mediators that can activate both eosinophils and gastric epithelial cells, in addition to triggering efficient eosinophil degranulation.

Transduction of* ICAM-1* shRNA-containing lentivirus into gastric epithelial cells just partially reduced the levels of released ECP (Figure 6(c)). These levels are pretty similar to those obtained by directly exposing eosinophils to 200 *μ*g/mL of purified OMVs (Figure 2(a)). Based on these results, it is conceivable that the ICAM-1 plays a more important role in regulating eosinophil recruitment, as previously reported [37], rather than in modulating eosinophil degranulation.

Since it has been previously reported that there is no evidence of OMV-associated CagA on vesicles shed by Cag-PAI^+^ strain 60190 [38], it would be conceivable that the absence of differences in terms of ECP levels observed by using a CagA-negative mutant strain could simply reflect the absence of CagA in OMV preparation. Considering that the most abundant of the eosinophil granule-derived proteins is MBP [39], an additional evaluation of MBP secretion would be more appropriate and informative and further studies are needed to evaluate the other eosinophil granule-derived proteins in eosinophils stimulated with OMVs. In the present study, it was not clear if the patients with gastric cancer were* H. pylori*-positive or* H. pylori*-negative. It is possible that* H. pylori* status might affect the results. Therefore, a limitation of our study is that we did not have any information regarding the histological evaluation of the apparently normal mucosa used for the isolation of primary epithelial cells.

Previous report implicates the cag pathogenicity island as a necessary factor for recruitment of eosinophils and found that loss of VacA increased eosinophil migration* in vitro* [6]. These results are concordant with further studies demonstrating that VacA may counteract effects of the cag islands in host cells [40, 41]. Based on these reports and the present study, it would be conceivable that VacA can downregulate CagA's effects on epithelial cells.

Our study used pharmacologic doses of OMVs to induce degranulation in eosinophils. Since the* in vitro* pharmacologic dose used in the present study is presumed to be higher than the physiologic concentration in gastric mucosa infected with* H. pylori*, further studies are needed to investigate the precise relationship between* in vitro* pharmacologic doses and the* in vivo* physiologic concentrations of OMVs.

In summary, here we demonstrated that exposure of eosinophils to OMVs, gastric epithelial cells prestimulated with OMVs, or gastric epithelial cells prestimulated with OMV-CM results in excessive degranulation. Moreover, eosinophil degranulation was induced via an ICAM-1- and CD11/CD18-dependent pathway. Based on these findings, we propose that the induction of eosinophil degranulation by* H. pylori* OMVs may be carried out through one of three processes: (1) stimulation of eosinophils by OMVs released from* H. pylori*, (2) direct contact of eosinophils with OMV-exposed gastric epithelial cells, or (3) direct contact of eosinophils with bystander gastric epithelial cells that have been stimulated with soluble mediators released from neighboring gastric epithelial cells exposed to OMVs.
